# Occupational exposure to human *Mycobacterium bovis* infection: A systematic review

**DOI:** 10.1371/journal.pntd.0006208

**Published:** 2018-01-16

**Authors:** Flora Vayr, Guillaume Martin-Blondel, Frederic Savall, Jean-Marc Soulat, Gaëtan Deffontaines, Fabrice Herin

**Affiliations:** 1 Service des Maladies Professionnelles et Environnementales, Centre Hospitalier Universitaire de Toulouse, Toulouse, France; 2 Département des maladies infectieuses et tropicales, Centre Hospitalier Universitaire de Toulouse, Toulouse, France; 3 Institut National de la Santé et de la Recherche Médicale, Unité Mixte de Recherche 1043 –Centre National de la Recherche Scientifique, Unité Mixte de Recherche 5282, Centre de Physiopathologie Toulouse-Purpan, Université Paul Sabatier Toulouse 3, Toulouse, France; 4 Laboratoire d’Anthropologie Moléculaire et imagerie de synthèse, Centre National de la Recherche Scientifique Unité mixte de recherche 5288, Université Paul Sabatier Toulouse 3, Toulouse, France; 5 Service de Médecine Légale, Centre Hospitalier Universitaire de Toulouse, Toulouse, France; 6 Laboratoire d’épidémiologie et analyses en santé publique, Institut National de la Santé et de la Recherche Médicale-Unité Mixte de Recherche 1027, Université Paul Sabatier Toulouse 3, Toulouse, France; 7 Caisse Centrale de la Mutualité Sociale Agricole, Les Mercuriales, Bagnolet, France; Swiss Tropical and Public Health Institute, SWITZERLAND

## Abstract

**Background:**

*Mycobacterium bovis (M*. *bovis)* is the main causative agent of bovine zoonotic tuberculosis. The aim of this systematic review is to highlight the occupational exposure to bovine tuberculosis due to *M*. *bovis*.

**Methodology/principal findings:**

A computer based literature search was carried out to identify papers published between January 2006 and March 2017. “PubMed, Cochrane Library and Science Direct” databases were searched systematically. Articles presenting the following properties were included: (i) focusing on *M*. *bovis*; (ii) concerning occupational exposure to bovine tuberculosis. A quality assessment was performed after selection of studies. Our search strategy identified a total of 3,264 papers of which 29 studies met the inclusion criteria. Of the 29 articles, 17 were cross-sectional studies (6 were of high quality and scored in the range of 6–7, 11 were of moderate quality and scored in the range 3–5), 10 were case reports, and 2 were reviews. Different occupational fields exposing to the disease were described: livestock sector, particularly in contact with dairy cattle (farmers, veterinaries and assistants, abattoir workers) and working in contact with wildlife (hunters, taxidermists).

**Conclusions:**

A specific guideline for occupational practitioners taking care of employees exposed to bovine tuberculosis is warranted and should be tailored to level of exposure. This review was intended to be the first step of such a project. Articles were identified from various continents and countries with different socio-economic situations, broadening our understanding of the worldwide situation. Published data on occupational exposure in developed countries are scarce. We had to extrapolate findings from countries with higher prevalence of the disease.

## Author summary

*Mycobacterium tuberculosis* complex causes tuberculosis, a widespread burden for humans, and other mammals. It includes *Mycobacterium bovis* which is responsible for zoonotic tuberculosis. Bovine tuberculosis infection in human historically occurs following consumption of unpasteurised dairy products. However, transmission via occupational exposure may also occur through inhalation of aerosols exhaled by infected animals or humans, and from direct contacts often associated with the presence of a wound. In this systematic review, we were able to gather data referring to occupational exposure, and precise different risk factors for transmission of the disease. We conclude that this knowledge can lead to the development of preventive measures at workstation to control the disease. A specific guideline for practitioners taking care of employees exposed to bovine tuberculosis is warranted and should be tailored to level of exposure.

## Introduction

Bovine tuberculosis might appear to be a minor topic in terms of public health, at least in industrialized countries, as human *Mycobacterium bovis (M*. *bovis)* infection accounts for a small proportion (0.5–7.2%) of all patients with a bacteriologically confirmed diagnosis of tuberculosis [1]. In developing countries, *M*. *bovis* infection probably still constitutes a major threat to public health [2]. The virulence of *M*. *bovis* has also generally been considered to be attenuated compared to *M*. *tuberculosis*, as defined by decreased transmissibility as well as being associated with a lower risk of human disease after infection [3–6].

Bovine tuberculosis infection in humans primarily and historically occurs following consumption of unpasteurised dairy products and close contacts with infected cattle [7]. Transmission mostly occurs via the gastrointestinal tract following consumption of contaminated dairy products [1,8], or to a lesser extent, contaminated meat [1,9]. Transmission via inhalation of aerosols exhaled by infected animals or humans or from direct contacts often associated with the presence of a wound may also occur [1]. Widespread milk pasteurisation and strict hygiene measures in livestock management have gradually reduced transmission of bovine tuberculosis in developed countries [1]. However, the risk globally persists, certainly more acutely in certain occupational categories, particularly in the livestock sector.

The main objective of this systematic review was to identify studies reporting and assessing occupational exposure to bovine tuberculosis.

## Methods

The PRISMA statement (Preferred Reported Items for Systematic Reviews and Meta-Analyses) was followed as a formal guideline for this review.

### Search strategy

This review of the literature included original papers published in peer-reviewed journals between January 2006 and March 2017. The search strategy queried the following databases: PubMed, Science Direct, and Cochrane Library, using an extensive keyword search: “bovine tuberculosis”, “zoonotic tuberculosis”, “*Mycobacterium bovis*”. An overview of search terms is shown in Additional file 1 (S1 Appendix).

### Selection of studies

Article titles identified during the initial search were first screened by two independent reviewers–the primary reviewer and the second reviewer. Articles selected on the basis of their title were then re-examined in the abstract review stage during which two reviewers independently assessed each abstract. During the third stage, full text papers considered to be relevant based on analysis of their abstracts were obtained and further evaluated by two reviewers in terms of relevancy, quality and inclusion/exclusion criteria. When the initial reviewers disagreed on inclusion of a study, a third reviewer was responsible for the final decision. In addition, reference lists of selected articles were further reviewed to find other relevant studies, particularly those not identified during the initial search.

### Inclusion criteria

Articles presenting the following properties were included: (i) focusing on *M*. *bovis*; (ii) concerning occupational exposure to bovine tuberculosis. Full inclusion criteria are shown in Additional File 2 (S2 Appendix).

### Exclusion criteria

Articles presenting the following properties were excluded: (i) concerning other mycobacteria (*M*. *tuberculosis*, *M*. *caprae*, *M*. *pinnipedii*, etc.) (ii) reporting epidemiological data on animals; (iii) focused on Genetics, Immunology, Microbiology, drug therapy or vaccination. Full exclusion criteria are shown in Additional file 2 (S2 Appendix).

### Type of studies

All types of articles were included: original articles using quantitative or qualitative data, case reports, protocols, reviews, and meta-analyses. Opinion articles and Editorials were excluded.

### Type of outcomes

Data were collected for identification of occupational exposure, predictors of transmission, and high-risk groups likely to develop the disease when exposed to *M*. *bovis*.

### Data extraction and quality assessment

Data were extracted from the papers included by one reviewer and were checked for accuracy by the second reviewer. Disagreement concerning data extraction between reviewers was resolved by consensus. Extracted data included: first author; year of publication; journal of publication; country; study design; study population; prevalence of bovine tuberculosis in humans, factors associated with *M*. *bovis* transmission to humans, including the presence or absence of an occupational context. Different quality assessment tools were used for qualitative and quantitative studies. The British Sociological Association (BSA) Medical Sociology Group was used for quality assessment of cross-sectional studies [10], the Quality Assessment Tool for Systematic Reviews of Observational Studies (QATSO) was used for the quality assessment of reviews [11], and finally the CARE (Case Report) checklist was used for the quality assessment of case reports [12]. More details about these quality assessments are provided in Additional file 3 (S3 Appendix).

## Results

Our search strategy identified a total of 3,264 articles; 318 duplicate articles were excluded and 113 full-text articles were assessed for eligibility. Following a thorough review, 29 articles were included in quality assessment and data synthesis (S1 PRISMA flowchart). Detailed findings of these articles are shown in Table 1. The background characteristics (study design, participants, reported prevalence of the disease, reported predictors of transmission of the disease to human, etc.) identified in these articles are shown in Table 1. Ten of the 29 studies were from Europe [1,9,13–20], four from America [21–24], eight from Africa [25–32], two from Asia [33,34], two from New Zealand [35,36], one from Australia [37] and two from the Middle East [38,39]. One study was based on worldwide data (review) [40]. Of the 29 articles, 17 were cross-sectional studies (6 were of high quality and scored in the range of 6–7, 11 were of moderate quality and scored in the range 3–5), 10 were case reports, and 2 were reviews.

**Table 1:** Studies describing occupational context of *M*. *bovis* human infection.

Study	Country	Participants / Sample	Study Design	Prevalence of the disease	Predictors of transmission
Adesokan HK *et al*, 2012, Int J Tuberc Lung Dis [29]	Nigeria	70 livestock traders	Cross-sectional	Overall, 10% (7/70) of livestock traders had a positive culture of sputum samples indicative of *M*. *tuberculosis* complex, which were differentiated into *M*. *bovis* (n = 2) and *M*. *tuberculosis* (n = 5) using deletion typing	Not described
Allix-Béguec C *et al*, 2010 Eur Respir J [9]	Belgium	70-year-old female living on a farm	Case report	Not described	She was a relative of a cattle breeder
Al-Thwani AN *et al*, 2016, Int J Mycobacteriol [38]	Iraq	186 workers who were in contact with slaughtered cattle	Cross-sectional	Three isolates were obtained from sputum samples of workers (1.6%); two of these isolates were diagnosed as *M*. *bovis* and the third as *M*. *tuberculosis*	Not described
Ameni G *et al*, 2013, PLoS One [26]	Ethiopia	287 households (146 households with a case of pulmonary tuberculosis and 141 households free of tuberculosis) and 287 herds	Cross-sectional	Herd prevalence of tuberculin test reactors was 9.4% and was higher (p<0.01) in herds owned by households with tuberculosis than in herds owned by tuberculosis-free households	Not described
Baker MG *et al*, 2006, Epidemiol Infect [36]	New Zealand	Not described	Cross-sectional	In New Zealand, 2.7% of all cases of tuberculosis are due to *M*. *bovis*. 54 patients infected with *M*. *bovis*	Risk factors for *M*. *bovis* infection: male, > 60 years old. Identified exposures were: living or working on a farm, being an abattoir worker, consumption of unpasteurized dairy products, veterinarian assistants, necropsies of wild animals
Bilal S *et al*, 2010, J Med Microbiol [13]	Ireland	Case 1: a 50-year old male, presumptive case of *M*. *bovis* infection. Case 2: a 35-year old female with bovine tuberculosis (confirmed case)	Case report	Not described	Case 1: farm worker. Case 2: history of contact with *M*. *bovis* at her household farm. No other risk factor identified
Chan HHY *et al*, 2015, N Z Med J [35]	New Zealand	50-year-old immunocompetent female with pulmonary tuberculosis caused by *M*. *bovis*	Case report	Not described	She had been employed for the last 7 years at the local freezing works, specifically working on the offal floor where animal organs (mainly beef) were cleaned and packed
Cleaveland S *et al*, 2007, Tuberculosis (Edinb) [27]	Tanzania	10549 cattle, 622 herds tested. Questionnaire for 239 households (living on the farm)	Cross-sectional	7 of 65 (10.8%) cases of human cervical adenitis due to *M*. *bovis*	Not described
Cordova E *et al*, 2012, Int J Tuberc Lung Dis [24]	Argentina	Retrospective analysis of patients with confirmed *M*. *bovis* infection between 1996 and 2008	Cross-sectional	N = 39 patients included, accounting for 0.4% of tuberculosis cases	93% of 39 patients had at least one risk factor: 65% had occupational exposure, 31% had a history of living in a rural area and 4% consumed unpasteurised milk
De La Rua-Domenech R *et al*, 2006, Tuberculosis [1]	United Kingdom	NA	Review	Not described	Review of bovine tuberculosis in the United Kingdom. Proposed guidelines
Gumi B *et al*, 2012, Ecohealth [28]	Ethiopia	260 Ethiopian pastoralists with suspected pulmonary tuberculosis and 32 with suspected lymphadenitis. In parallel, 207 suspected tuberculous lesions from cattle, goats and camels at abattoirs	Cross-sectional	3 out of 173 human isolates were identified as *M*. *bovis*	Not described
Hambolu D *et al*, 2013, PLoS One [29]	Nigeria	349 randomly selected meat handlers in an abattoir	Cross-sectional	Not described	Risk was linked with eating “Fuku Elegusi” (eating the visibly infected parts of the lung of cattle in order to convince customers to buy the meat). Prevalence of this technique was 22% among employees
Ingram PR *et al*, 2010, Commun Dis Intell Q Rep [37]	Australia	52-year-old male with pulmonary tuberculosis due to *M*. *bovis*	Case report	Not described	Patient had worked as a butcher for the past 35 years. He recalled slaughtering animals suspected to have bovine tuberculosis several decades ago. This process was accompanied by dissection of the diseased lungs
Jalava K *et al*, 2007, Epidemiol Infect [19]	United Kingdom	A case was defined as a culture-positive *M*. *bovis* case	Cross-sectional	A total of 315 *M*. *bovis* cases in humans were identified in England, Wales and Northern Ireland between 1993 and 2003	Where information was available, 49% (n = 41) of cases reported consumption of unpasteurised dairy products and 46% (n = 30) had occupational contact with cattle. Contact with a farm was reported for 39 (48%) cases and 24 (22%) had contact with a human tuberculosis case
Khattak I *et al*, 2016, Occup Med (London) [33]	Pakistan	A total of 141 abattoir workers, 317 butchers, 50 livestock farmers, five veterinary doctors and three veterinary assistants took part of the study	Cross-sectional	Four out of 16 abattoir workers with chronic cough from whom sputum samples were obtained and 1 out of 50 livestock farmers were positive for *M*. *bovis* by PCR analysis of sputum samples. Duration of work as an abattoir worker was significantly associated (P < 0.05) with prevalence of zoonotic tuberculosis. The knowledge of abattoir workers, butchers, livestock farmers and veterinary assistants regarding transmission of bovine tuberculosis from animals to humans and the symptoms of tuberculosis in humans was very poor	Not described
Larsen MV *et al*, 2008, Eur Respir J [14]	Denmark	A 79-year-old female with a history of severe erosive seropositive rheumatoid arthritis and reactivation of bovine tuberculosis (ascites)	Case report	Not described	She had worked at a dairy, with probable exposure to unpasteurised milk from *M*. *bovis*-infected cattle. Reactivation of the disease after treatment with tumour necrosis factor α inhibitors
Lassausaie J *et al*, 2015, Epidemiol Infect [34]	Laos	80 elephants working and 142 mahouts or owners	Cross-sectional	36% of the elephants were seroreactive to the test, no human participant was smear- or culture-positive for *M*. *bovis*	Not described
Mertoğlu A *et al*, 2016, Clin Respi J [39]	Turkey	46-year-old male patient with cutaneous (non-healing wound on his hand) and pulmonary tuberculosis	Case report	Not described	Butcher who had been working in a slaughterhouse
Nuru A *et al*, 2017, BMC Res Notes [30]	Ethiopia	70 cases of human tuberculous lymphadenitis among smallholder farmers	Cross-sectional	Positive cultures of tuberculosis in 40 of the 70 cases, 2 isolates of *M*. *bovis*	65.7% (46/70) of the respondents were not aware of zoonotic tuberculosis, and 67.1% (47/70) of them drank raw milk
Oloya J *et al*, 2008, Epidemiol Infect [31]	Uganda	Lymph node biopsies (n = 43) of patients with cervical lymphadenitis reporting for tuberculosis treatment in Matany and Moroto Hospitals in the transhumant areas of Karamoja, Uganda	Cross-sectional	*M*. *bovis* was isolated on 3 of the 43 biopsies	Not described
Rodriguez E *et al*, 2009, Int J Tuberc Lung Dis [20]	Spain	Retrospective study covering all *M*. *bovis* and *M*. *caprae* isolates identified at the National Mycobacterial Reference Laboratory (NRL) from 2004–2007	Cross-sectional	The study covered 110 isolates (89 *M*. *bovis* and 21 *M*. *caprae*) that accounted for 1.9% and 0.3% of *M*. *tuberculosis* complex isolates available at the NRL, respectively	Data on risk exposure were available in 82 (74%) of the 110 cases, with 60 registering a probable (occupational exposure (crop and livestock farmers)) or possible (patients born in countries with a high prevalence of bovine tuberculosis) risk of exposure and 22 registering no risk
Shrikrishna D *et al*, 2009, Thorax [15]	United Kingdom	Case 1: a 42-year-old female with bovine tuberculosis; Case 2: latent tuberculous infection of her 12-year-old daughter	Case report	Not described	Potential occupational exposure to *M*. *bovis*. Veterinary nurse for two local practices during 4 years. Assistance for tuberculin tests of cattle herds (2 tests reactors had positive culture for *M*. *bovis* at post mortem). The patient recalled picking up an injured badger on the road
Sunder S *et al*, 2009, Journal of Clinical Microbiology [16]	France	Case 1: a 50-year-old man born in France; Case 2: his 20-year-old daughter	Case report	Not described	Case 1: Occupational exposure (abattoir worker, handling carcasses and offal of contaminated animals). Case 2: Intra-familial transmission, proven by spoligotyping (same strains)
Tebug SF *et al*, 2014, Onderstepoort J Vet Res [32]	Malawi	140 out of 684 registered dairy farmers	Cross-sectional	Not described	Almost all survey participants (96.4%) practiced at least one farm activity that could lead to transmission of bovine tuberculosis, including sale (67.0%) and consumption (34.0%) of unpasteurised milk
Thoen C *et al*, 2006, Vet Microbiol [40]	International	N = 237 articles	Review	Not described	Risk factors: consumption of infected milk, meat industry and working in slaughterhouses in regions where the infection is prevalent in cattle. Evidence of person-to-person transmission is rare. In industrialized countries: epizootics in domesticated and wild mammals and latent infection in immigrants
Torres-Gonzalez P *et al*, *2013*, PLoS Negl Trop Dis [21]	Mexico	Tuberculin skin test and IGRA performed in 311 dairy farm and abattoir workers and their household contacts. Sputa were collected from individuals with respiratory symptoms	Cross-sectional	Prevalence of latent tuberculosis infection: 76.2% (95% CI: 71.4–80.9%) by tuberculin skin test and 58.5% (95% CI: 53–64%) by IGRA. 2 subjects diagnosed with pulmonary tuberculosis caused by *M*. *bovis*	3 categories of exposure were defined. High risk (direct contact with livestock in closed spaces): abattoir workers, veterinary personal performing cattle necropsies, foremen, milkers. Medium risk (direct contact with livestock in open spaces): tractor operators, breeders, feeders, other veterinary personal, maintenance technicians, household contacts living in the cowshed. Low risk (no direct contact with livestock): owners of the cowshed, administrative clerks, people involved in commercial activities
Twomey DF *et al*, *2011*, Vet Microbiol [17]	United Kingdom	A 25-year-old female, BCG-vaccinated, veterinary surgeon	Case report	Not described	Cutaneous bovine tuberculosis (*M*. *bovis*) following contact with an infected Alpaca. Exposure: several examinations of the infected alpaca, thoracocentesis of the animal, euthanasia with intravenous injection, examination of the carcass after death. Probable infection pathway: no gloves while euthanizing the animal and accidental contamination of the surgeon's hands with blood at the time of venipuncture. Tuberculous lesion of her thumb
Wilkins MJ *et al*, 2008, Emerg Infect Dis [22]	United States	Case 1: a-74-year-old man with bovine tuberculosis; Case 2: a 29-year-old man	Case report	Not described	Case 1: Hunting area: business with a buck pole where hunters displayed killed deer, hunting white-tailed deer and consuming venison, handling a deer carcass and recreational feeding of deer. Case 2: while field dressing a white-tailed deer, he punctured his left finger with a hunting knife
Wilkins MJ *et al*, 2009, Prev Vet Med [23]	United States	Veterinarians (n = 259) who had completed at least five official bovine tuberculosis herd tests in Michigan in 2001	Cross-sectional	Thirty-six veterinarians reported a total of 53 injuries (10 major, 12 minor and 31 self-treated). Hands (29%) and legs (21%) were the anatomical sites most frequently injured, and sprains/strains (30%) and abrasion/contusion (30%) were the most common types of injuries sustained	Not described

The main forms of occupational exposure to *M*. *bovis* are shown in Table 2.

**Table 2:** Overview of occupational exposure to *M*. *bovis*.

Occupational exposure	Transmission pathways	Preventive measures
Farmers	Respiratory transmission (close contacts with cows)	Herd testing, hygiene measures and respiratory protection if an animal has respiratory symptoms
Veterinarians and assistants	Accidental cutaneous inoculation (wound) and possible respiratory transmission while performing necropsies	Wearing gloves and respiratory protection while performing medical procedures involving close contacts with infected animals
Slaughterhouse workers	Accidental cutaneous inoculation while manipulating carcasses with knives (wound)	Hygiene measures. Wearing gloves. Information about clinical signs suggestive of bovine tuberculosis, transmission pathways of the disease and management of infected carcasses
Hunters and workers with wild animals		

In all groups, consumption of unpasteurised dairy products is a known transmission pathway. Immunosuppression increases the risk of reactivation of latent bovine tuberculosis.

### Livestock farmers

Our review reveals an over-representation of livestock farming in the occupational exposure to *M*. *bovis*, and particularly working or living with cattle. One third of studies (excluding reviews) specifically reported exposure to livestock [9,21,25–28,30–32]. This assumption could, however, be associated with a publication bias as no study has ever been designed to formally compare different occupational exposures. In Mexico, among 311 dairy farmers, abattoir workers, and their household contacts, the prevalence of latent tuberculosis infection assessed by tuberculin skin test (TST) or Interferon Gamma Released Assay (IGRA) was 76.2% (95% CI: 71.4–80.9%), and 58.5% (95% CI: 53–64%), respectively [21]. Two subjects were diagnosed with *M*. *bovis*-related pulmonary tuberculosis, including one case genetically linked to animal infection. The prevalence of latent tuberculosis infection in this study was higher than in other populations in Mexico, and was strongly associated with occupational exposure (OR 2.72; 95% CI: 1.31–5.64), suggesting a link with *M*. *bovis*. Furthermore, the prevalence of symptomatic bovine tuberculosis appears to be higher in the population of livestock farmers than in the general population. In Nigeria, 10% (7/70) of livestock farmers had positive sputum culture indicative of *M*. *tuberculosis* complex, which were differentiated into *M*. *bovis* (n = 2) and *M*. *tuberculosis* (n = 5) using deletion typing [27]. Among 70 farmers with tuberculous lymphadenitis in Ethiopia, 40 had a positive culture for tuberculosis and 2 isolates were positive for *M*. *bovis* [30]. Finally, among 43 patients with cervical lymphadenitis in Uganda, 3 biopsies were positive for *M*. *bovis* [31]. All these studies report a significant prevalence of *M*. *bovis* infection among occupationally exposed livestock herders.

Cattle were the main reservoir of infection in most studies, although *M*. *bovis* can also infect goats and other dairy animals [1]. No study has described the risk of infection for meat production livestock farmers, suggesting a higher risk for dairy herders. A lack of knowledge concerning bovine tuberculosis, its pathways of transmission and prevention have been described as risk factors for infection [33].

### Veterinarians and assistants

Veterinarians and their assistants, occupationally related to farmers, are also exposed to *M*. *bovis* infection. The pathway of transmission may be airborne while performing respiratory examinations or necropsies. Cutaneous transmission has also been described, often associated with skin wounds that are frequent injuries in this occupation. One study describing the most common types of injury experienced by veterinarians showed that abrasions and contusions represented 30% of all injuries [23]. Two case reports illustrate this pathway of transmission. A 25-year-old female veterinary surgeon developed *M*. *bovis* cutaneous tuberculosis after being exposed to an infected Alpaca [17]. The authors explained that she was probably infected while euthanizing the animal without gloves and after accidental contamination with infected blood at the time of venipuncture. She did not report any wounds on her hands. The tuberculous lesion was localized on her thumb. Another case concerned a 42-year-old female potentially infected when she was a veterinary nurse performing tuberculin tests in herds of cattle [15]. Two of the animals that she had tested had positive cultures for *M*. *bovis* at post-mortem examination. The authors were unable to conclude on the pathway of transmission, as she also reported potential exposure to *M*. *bovis* while picking up an injured badger on the road.

### Abattoir workers

Abattoir workers are also exposed, as they manipulate infected carcasses and also use knives, increasing their risk of occupational injuries. In Pakistan, 4 out of 16 abattoir workers and one out of 50 livestock farmers with chronic cough were positive for *M*. *bovis* by PCR (polymerase chain reaction) analysis of sputum samples [33]. In this study, the duration of work as an abattoir worker was significantly associated with the prevalence of zoonotic tuberculosis. In 2015, a 50-year-old immunocompetent female with *M*. *bovis* pulmonary tuberculosis was probably infected as a result of occupational exposure [35]. She had been employed for at least seven years at the local freezing works, specifically working on the offal floor, where animal organs (beef) were cleaned and packed. In Turkey, a 46-year-old male former butcher working in a slaughterhouse, presented a non-healing wound on his hand [39]. He was diagnosed with *M*. *bovis* pulmonary and cutaneous tuberculosis. Finally, another case report illustrated this pathway of transmission, describing an abattoir worker who handled carcasses and offal of contaminated animals [16].

### Hunters and wildlife

Occupational exposure has also been described for hunters and workers in contact with wildlife. A review of the literature revealed two cases. A 74-year-old man whose risk factors were working in hunting zone comprising a buck pole where hunters displayed killed deer, hunting white-tailed deer and consuming venison, handling deer carcasses, and recreational feeding on deer [22]. The second case was a 29-year-old man who punctured his left finger with a hunting knife while field dressing a white-tailed deer. The authors identified exposed occupational categories as hunters, trappers, taxidermists, venison producers and venison consumers [22].

### High-risk groups

Finally, three categories of exposure risk were proposed by Torres-Gonzalez *et al* using immunological tests for latent tuberculosis infection in Mexico [21]. High risk (direct contact with livestock in closed spaces) concerns abattoir workers, veterinary personnel performing cattle necropsies, foremen, and milkers. Medium risk (direct contact with livestock in open spaces) concerns tractor operators, herders, feeders, other veterinary personnel, maintenance technicians, and household contacts living in the cowshed. Finally, low risk (no direct contact with livestock) concerns cowshed owners, administrative clerks, and people involved in commercial activities.

## Discussion

Ending the global TB epidemic, including zoonotic tuberculosis, is one of the objectives of the United Nations Sustainable Development Goals (SDGs), which have set the stage for multidisciplinary approaches to improve global health throughout the world by 2030 [41–42]. In 2014, the World Health Organization (WHO) defined the End TB Strategy, a resolution which calls on governments to adapt and implement a strategy with high-level commitment and financing [43]. The fourth edition of the Stop TB Partnership’s Global Plan to End TB 2016-2020-The Paradigm Shift was the first to highlight people at risk for zoonotic TB as a neglected population deserving greater attention [41,44]. The scope of our review addresses some of the priority areas identified for tackling zoonotic tuberculosis in the “Roadmap for zoonotic tuberculosis” published in 2017 by World Health Organization (WHO), Food and Agriculture Organization of the United Nations (FAO) and World Organization for Animal Health (OIE). Our review is consistent with one of the ten priorities proposed in their publication, which is to reduce the risk to people by identifying key populations and risk pathways for transmission of zoonotic TB. The roadmap highlights the need to define groups at risk of disease, including people with an occupational exposure [41].

Various occupational fields exposed to *M*. *bovis* infection can be distinguished: working with livestock, particularly dairy cattle (farmers, veterinarians and assistants, abattoir workers) and working in contact with wildlife (hunters, taxidermists). Studies have been conducted in developing countries with a heavy burden of zoonotic tuberculosis to assess the prevalence of infected farmers or abattoir workers. However, to our knowledge, this is the first review to identify the various occupational categories exposed to bovine tuberculosis.

Although the estimated prevalence of bovine tuberculosis in each region of the world is relatively low at the present time, the true incidence of zoonotic tuberculosis remains uncertain because of the absence of routine surveillance data from most countries [45–46]. Identification and differentiation of *M*. *tuberculosis* and *M*. *bovis* is not systematically performed. However, this differential diagnosis is crucial, as *M*. *bovis* is naturally resistant to pyrazinamide, one of the four medications used in the standard first-line anti-tuberculosis treatment regimen. Identification of risk factors for *M*. *bovis* infection is therefore extremely relevant for physicians managing patients with tuberculosis.

Risks were globally increased in developing countries with a higher prevalence of the disease. Combined with a lack of veterinary health control designed to limit *M*. *bovis* infection in herds, a wide range of breeding practices in developing countries could explain this increased risk, including proximity between human houses and animal shelters, shared material between farmers without disinfection precautions, consumption of unpasteurised dairy products and milk by farmers, their family and their customers, regular physical contacts with animals, lack of knowledge concerning the disease and its pathways of transmission, lack of hygiene practices, and finally proximity between cattle and wildlife [9,21,25–28,30–32].

This review of the literature revealed that only limited data are available concerning occupational exposure to bovine tuberculosis. Assessment of the concerned tasks or high-risk occupational groups was limited, particularly in industrialized countries. Pathways of transmission in occupational context were respiratory, cutaneous, often associated with wounds, and, to a lesser extent, digestive associated with lifestyle practices (consumption of unpasteurised dairy products by farmers). Cattle were the main reservoir of *M*. *bovis* infection.

Our study has a number of strengths. Our systematic review is based on several scientifically validated databases. The study methodology complied with the Preferred Reporting Items for Systematic Reviews and Meta-analyses (PRISMA) statement. We used validated quality assessment tools for each article. Articles were identified from various continents and countries with different socio-economic situations, broadening our understanding of the worldwide situation. Our limited search period ensured a maximum of recent knowledge. In view of the limited volume of published data, we were able to use key words without limiting the search, thereby limiting the risk of missing important articles. The literature search identified case reports, allowing a better understanding of how employees may be exposed to *M*. *bovis*. Regardless of the limited value of case reports in proving direct evidence for medical practice, they may strengthen our knowledge of human *M*. *bovis* infection and lead to the development of preventive measures to control the disease. We identified risk factors for transmission, allowing prevention to be targeted to employees most likely to develop this disease.

However, our study also has a number of limitations. First, published data on occupational exposure in developed countries are scarce. We had to extrapolate findings from countries with different prevalences of the disease, challenges, socio-economic situations, and lifestyles. Second, a number of articles were not available for review despite university access to databases. However, this bias was limited by the limited number of articles excluded. Third, we did not investigate exposure of animal caregivers in zoos and aquariums because no relevant article, meeting the inclusion/exclusion criteria, was found on this subject. Data gathered in this review gives the opportunity to expose transmission routes and risk profiles associated with occupational exposure. The narrow breadth of data available on this topic however limits the weight of our conclusions that should thus be interpreted with caution. Bovine tuberculosis is not a new disease, but it has long been neglected. As a result, the available information is mostly based on subnational data gathered from a limited number of countries, most of which are high-income with a low burden of disease in people and livestock. [41] Although our review was originally designed to gather studies without any geographical limitation in order to present a global outlook of the occupational exposure risk, our analysis is mainly relevant for the Western world, as we considered work organization of high-income countries. Any generalization of our findings to other parts of the world, where true exposure dynamics are far more complicated, may be associated with bias and should incorporate other factors and determinants that we could not evaluate in this analysis. Finally, another potential limitation could be associated with a publication bias related to external funding.

Future research should focus on assessing occupational exposure to bovine tuberculosis of different high-risk occupational categories in industrialized countries. A specific guideline for occupational practitioners taking care of employees exposed to bovine tuberculosis is warranted and should be tailored to level of exposure. This review was intended to be the first step of such a project. Combining expertise and efforts from different fields and institutions is crucial and will broaden the scope of options to address the challenges we still face today at the animal-human interface. Based on our review, no data on primary prevention of occupational exposure are available. The diagnosis and treatment of bovine tuberculosis are well known, but primary prevention modalities have yet to be defined. The occupational categories at risk and the main pathways of transmission highlighted in this review may help to design prevention messages for employees and prioritize information to immunosuppressed workers. As livestock farmers are mainly exposed to respiratory transmission by close contacts with cattle, prevention should focus on respiratory protection (mask) while working with infected animals with respiratory symptoms. Respiratory transmission should not be underestimated for veterinarians and their assistants, even though our review did not focus on this transmission route [47–48]. Cutaneous inoculation through a wound is a potential pathway of transmission for veterinarians and their assistants, slaughterhouse workers, and workers in close contact with wildlife. Gloves should be used while performing procedures on infected animals. Workers should receive information on the clinical signs of the disease, its pathways of transmission and how to manage a sick animal to reduce the risk of transmission. Consumption of unpasteurised dairy products remains a real risk in all groups. Transmission routes and risk profiles are likely to vary at continental level. Assessing the level of occupational exposure implies to consider the geographical, social, and economical settings in which the analysis is performed. In France, a guideline concerning medical follow-up of exposed employees should be published in the near future. Because of the interdependence between the health of people, animals and the environment, zoonotic TB in people cannot be fully addressed without controlling bovine TB in animals and improving food safety. A One Health approach is crucial to address this challenge.

## Supporting information

S1 PRISMA checklist (PDF)

S1 PRISMA flowchart (PDF)

S1 Appendix Extensive overview of search terms. (PDF)

S2 Appendix Inclusion and exclusion criteria for selection of articles. (PDF)

S3 Appendix Summary of the quality assessment tools. (PDF)
