# Supplementary material for: Occupational exposure to human Mycobacterium bovis infection: A systematic review
Source: PLoS Negl Trop Dis. 2018 Jan 16;12(1):e0006208. doi: 10.1371/journal.pntd.0006208 (PMC5786333; doi:10.1371/journal.pntd.0006208)
Supplement: S1 PRISMA flowchart — (PDF) [file pntd.0006208.s002.pdf]

PRISMA 2009 Flow Diagram

S4 Appendix. Flow chart

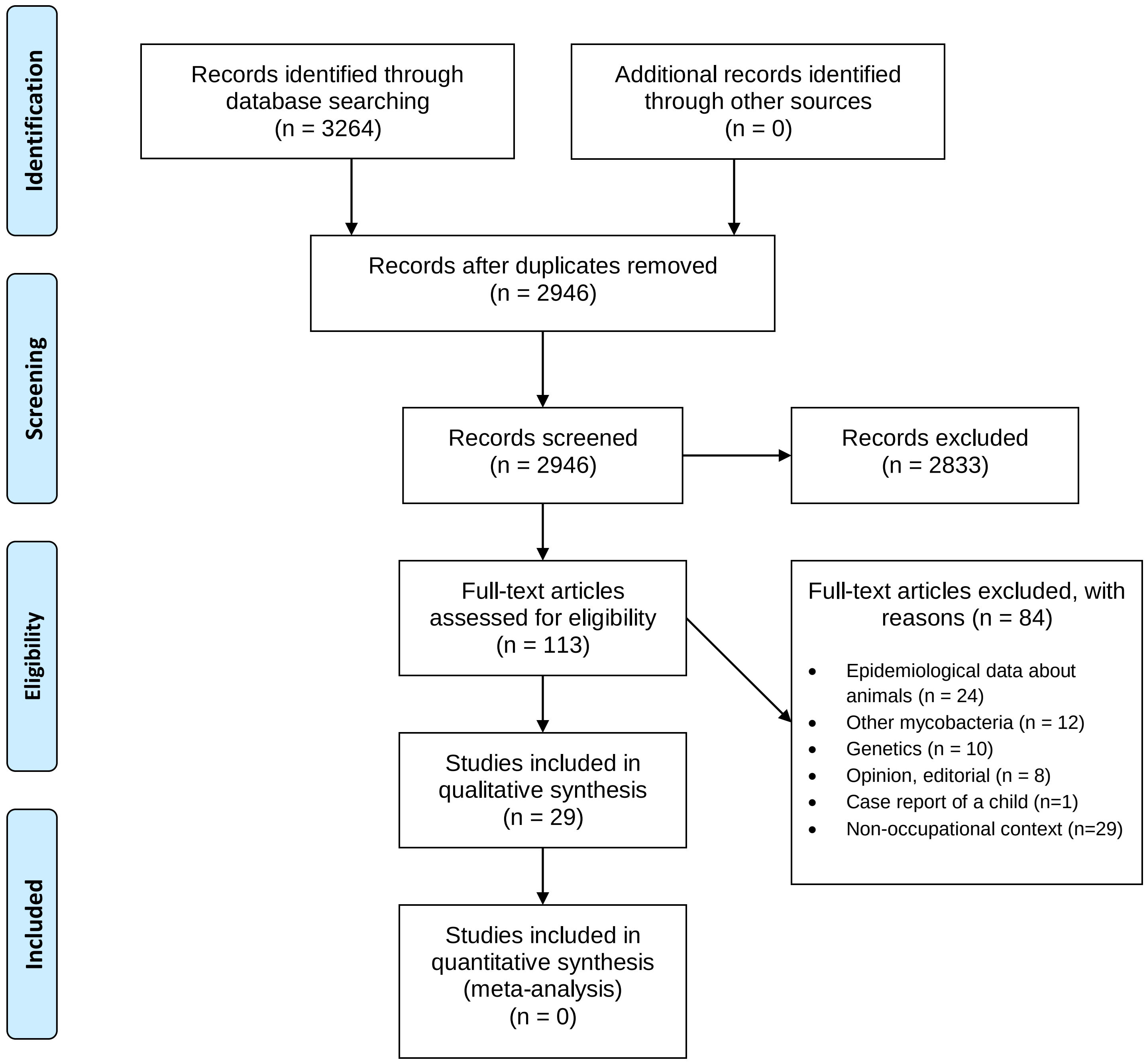

From: Moher D, Liberati A, Tetzlaff J, Altman DG, The PRISMA Group (2009). *P*referred *R*eporting *I*tems for *S*ystematic Reviews and *M*eta-*A*nalyses: The PRISMA Statement. PLoS Med 6(6): e1000097. doi:10.1371/journal.pmed1000097

For more information, visit www.prisma-statement.org.
