# Supplementary material for: Occupational exposure to human Mycobacterium bovis infection: A systematic review
Source: PLoS Negl Trop Dis. 2018 Jan 16;12(1):e0006208. doi: 10.1371/journal.pntd.0006208 (PMC5786333; doi:10.1371/journal.pntd.0006208)
Supplement: S1 Appendix — (PDF) [file pntd.0006208.s003.pdf]

S1 Appendix. Extensive overview of search terms

PubMed:

(Bovine tuberculosis) OR (zoonotic tuberculosis) OR (mycobacterium bovis)
Filter: human
Filter: From January 2006 to March 2017

("tuberculosis, bovine"[MeSH Terms] OR ("tuberculosis"[All Fields] AND "bovine"[All Fields]) OR "bovine tuberculosis"[All Fields] OR ("bovine"[All Fields] AND "tuberculosis"[All Fields])) OR (zoonotic [All Fields] AND ("tuberculosis"[MeSH Terms] OR "tuberculosis"[All Fields])) OR ("mycobacterium bovis"[MeSH Terms] OR ("mycobacterium"[All Fields] AND "bovis"[All Fields]) OR "mycobacterium bovis"[All Fields]) AND (("2006/01/01"[PDAT]: "2017/03/31"[PDAT]) AND "humans"[MeSH Terms])

Science direct:

(Bovine tuberculosis) OR (zoonotic tuberculosis) OR (mycobacterium bovis)
Filter: From 2006 to 2017
Filter (topics): patient / mycobacterium / tuberculosis / human / disease / infection
Filter: Journal

pub-date > 2005 and (zoonotic tuberculosis)OR(bovine tuberculosis)OR(mycobacterium bovis) AND LIMIT-TO(topics, "mycobacterium,patient,tuberculosis,human,disease,infection") .

Cochrane library:

(Bovine tuberculosis) OR (zoonotic tuberculosis) OR (mycobacterium bovis)
Filter: From 2006 to 2017

(Bovine tuberculosis) or (zoonotic tuberculosis) or (mycobacterium bovis):ti,ab,kw Publication Year from 2006 to 2017 (Word variations have been searched)
