# Supplementary material for: Occupational exposure to human Mycobacterium bovis infection: A systematic review
Source: PLoS Negl Trop Dis. 2018 Jan 16;12(1):e0006208. doi: 10.1371/journal.pntd.0006208 (PMC5786333; doi:10.1371/journal.pntd.0006208)
Supplement: S2 Appendix — (PDF) [file pntd.0006208.s004.pdf]

S2 Appendix. **Inclusion and exclusion criteria for selection of articles**

Inclusion criteria	Exclusion criteria
• Adults (≥ 18 years old)<br>• All countries<br>• From 2006 to 2017<br>• *Mycobacterium bovis*<br>• Occupational exposure to bovine tuberculosis	• Other mycobacteria<br>• Epidemiological data about animals<br>• Diagnosis of infection in animals<br>• Genetics<br>• Microbiology<br>• Immunology<br>• Molecular biology<br>• Diagnostic performance of tests<br>• Vaccination<br>• Therapeutics<br>• Type of article: opinion, editorial
