# Supplementary material for: Occupational exposure to human Mycobacterium bovis infection: A systematic review
Source: PLoS Negl Trop Dis. 2018 Jan 16;12(1):e0006208. doi: 10.1371/journal.pntd.0006208 (PMC5786333; doi:10.1371/journal.pntd.0006208)
Supplement: S3 Appendix — (PDF) [file pntd.0006208.s005.pdf]

## S3 Appendix. Summary of the Quality Assessment Tools

| *Study Design* | *Quality Assessment Tool* | *Quality Rating of study* |
|---|---|---|
| Cross-Sectional study | BSA Medical Sociology Group | Score 1-7<br>1-2 (Low)<br>3-5 (Moderate)<br>6-7 (High) |
| Longitudinal study | The Quality Assessment Tool for Systematic Reviews of Observational studies (QATSO) | External validity (1 item)<br>Reporting (2 items)<br>Bias (1 item)<br>Confounding (1 item) |
| Case report | The CARE checklist (2016) | /28 |

**Table**: Quality of included studies

| Author | Year and Journal of Publication | Study Design | Quality assessment tool | Quality Rating of study | Comments |
|---|---|---|---|---|---|
| **Occupational context** | | | | | |
| Adesokan HK *et al* | 2012, Int J Tuberc Lung Dis. | Cross-sectional study | BSA Medical sociology group | 4 | Moderate |
| Allix-Béguec C *et al* | 2010 Eur Respir J. | Case report | CARE checklist | 13/28 | |
| Al-Thwani AN *et al* | 2016, Int J Mycobacteriol | Cross-sectional study | BSA Medical sociology group | 4 | Moderate |
| Ameni G *et al* | 2013, PLoS One | Cross-sectional study | BSA Medical sociology group | 6 | High |
| Baker MG *et al* | 2006, Epidemiol Infect. | Cross-sectional study | BSA Medical sociology group | 5 | Moderate |
| Bilal S *et al* | 2010, J Med Microbiol | Case report | CARE checklist | 18/28 | |
| Chan HHY *et al* | 2015, N Z Med J | Case report | CARE checklist | 14/28 | |
| Cleaveland S *et al* | 2007, Tuberculosis (Edinb) | Cross-sectional study | BSA Medical sociology group | 6 | High |
| Cordova E *et al* | 2012, Int J Tuberc Lung Dis | Cross-sectional study | BSA Medical sociology group | 4 | Moderate |
| De La Rua-Domenech R *et al* | 2006, Tuberculosis | Review | The Quality Assessment Tool for Systematic Reviews of Observational studies (QATSO) | External validity 1<br>Reporting 1<br>Bias 0<br>Confounding 0 | Fair |
| Gumi B *et al* | 2012, Ecohealth | Cross-sectional study | BSA Medical sociology group | 5 | Moderate |
| Hambolu D *et al* | 2013, PLoS One | Cross-sectional study | BSA Medical sociology group | 6 | High |
| Ingram PR *et al* | 2010, Commun Dis Intell Q Rep | Case report | CARE checklist | 14/28 | |
| Jalava K *et al* | 2007, Epidemiol Infect | Cross-sectional study | BSA Medical sociology group | 5 | Moderate |
| Khattak I *et al* | 2016, Occup Med (London) | Cross-sectional study | BSA Medical sociology group | 4 | Moderate |
| Larsen MV *et al* | 2008, Eur Respir J | Case report | CARE checklist | 21/28 | |
| Lassausaie J *et al* | 2015, Epidemiol Infect | Cross-sectional study | BSA Medical sociology group | 4 | Moderate |
| Mertoğlu A *et al* | 2016, Clin Respir J | Case report | CARE checklist | 19/28 | |
| Nuru A *et al* | 2017, BMC Res Notes | Cross-sectional study | BSA Medical sociology group | 4 | Moderate |
| Oloya J *et al* | 2008, Epidemiol Infect | Cross-sectional study | BSA Medical sociology group | 4 | Moderate |
| Rodriguez E *et al* | 2009, Int J Tuberc Lung Dis | Cross-sectional study | BSA Medical sociology group | 6 | High |
| Shrikrishna D *et al* | 2009, Thorax | Case report | CARE checklist | 15/28 | |

| | | | | | |
|---|---|---|---|---|---|
| Sunder S *et al* | 2009, Journal of Clinical Microbiology | Case report | CARE checklist | 15/28 | |
| Tebug SF *et al* | 2014, Onderstepoort J Vet Res | Cross-sectional study | BSA Medical sociology group | 4 | Moderate |
| Thoen C *et al* | 2006, Vet Microbiol | Review | The Quality Assessment Tool for Systematic Reviews of Observational studies (QATSO) | External validity 1<br>Reporting 1<br>Bias 0<br>Confounding 0 | Poor |
| Torres-Gonzalez P *et al* | 2013, PLoS Negl Trop Dis. | Cross-sectional study | BSA Medical sociology group | 6 | High |
| Twomey DF *et al* | 2010, Vet Rec | Case report | CARE checklist | 11/28 | |
| Wilkins MJ *et al* | 2008, Emerg Infect Dis. | Case report | CARE checklist | 15/28 | |
| Wilkins MJ *et al* | 2009, Prev Vet Med | Cross-sectional study | BSA Medical sociology group | 6 | High |

QUALITY ASSESSMENT OF CROSS-SECTIONAL STUDIES (BSA Medical Sociology Group)

Seven Quality Indicators:
1) Appropriate Research Design
2) Appropriate Recruitment Strategy
3) Response Rate Reported
4) Sample Representative of Similar Population
5) Objective and Reliable Measures Used
6) Power Calculation/Justification of Numbers Reported
7) Appropriate Statistical Analysis

** Y=Yes N=No [ Quality Indicators Met out of 7: 1-2 (Low)---- 3-5 (Moderate)----6-7 (High) ]

| **Author and year** | **Appropriate Research Design? (Y/N)** | **Appropriate Recruitment Strategy? (Y/N)** | **Response Rate? (Y/N) %** | **Is Sample Representative? (All similar populations) (Y/N)** | **Objective and Reliable Measures ? (Y/N)** | **Power Calculation/ Justification of Numbers? (Y/N)** | **Appropriate Statistical Analysis? (Y/N)** | **Quality Indicators Met (out of 7)** |
|---|---|---|---|---|---|---|---|---|

## QUALITY ASSESSMENT OF LONGITUDINAL STUDIES

**The Quality Assessment Tool for Systematic Reviews of Observational Studies (QATSO) Score:**

Five item for quality indicator

**1) External validity (1 item)** – addresses the extent to which the findings from the study can be generalised to the population from which the study subjects are derived.
**2) Reporting (2 items)** – assesses whether the information provided in the paper is sufficient to allow a reader to make an unbiased assessment of the findings of the study. One of the items is specific for prevalence studies.
**3) Bias (1 item)** – addresses bias in the measurement of the outcomes in a study.
**4) Confounding (1 item)** – addresses whether studies have applied adjustment for confounding in the analysis. This item is specific to studies concerning association of risk factors.

Although the QATSO Score consists of five items, users may select 4–5 items depending on the type of studies being evaluated. Studies achieving 67% or more in the score will be regarded as "good" quality; 34–66% "fair"; and, below 33% "poor".

**The CARE (Case Report guidelines) Checklist (2016) of information to include when writing a case report**

| | | |
|---|---|---|
| **Title** | **1** | The words "case report" should be in the title along with the area of focus |
| **Key Words** | **2** | Four to seven key words—including "case report" as a key word |
| **Abstract** | **3a** | Background: What does this case report add to the medical literature |
| | **3b** | Case summary (1 paragraph): chief complaint, diagnoses, interventions, and outcomes |
| | **3c** | Conclusion: What are the main "take-away" lessons from this case? |
| **Introduction** | **4** | How does this case informs healthcare delivery—with references (1-2 paragraphs) |
| **Timeline** | **5** | Relevant information from this case report organized into a timeline (table or figure) |
| **Patient Information** | **6a** | De-identified demographic and other patient specific information |
| | **6b** | Chief complaint (what prompted this patient visit) |
| | **6c** | Relevant medical and psychosocial history (including interventions and outcomes) |
| **Physical Exam** | **7** | Relevant physical examination findings |
| **Diagnostic Assessment** | **8a** | Diagnostic evaluations (such as laboratory testing, imaging, surveys) |
| | **8b** | Diagnoses (consider tables/figures linking assessment with diagnoses and interventions) |
| | **8c** | Diagnostic reasoning including other diagnoses considered and diagnostic challenges |
| | **8d** | Prognostic characteristics (such as staging in oncology) where applicable |
| **Interventions** | **9a** | Types of intervention (such as pharmacologic, surgical, preventive, self-care) |
| | **9b** | Intervention administration (such as dosage, strength, duration) |
| | **9c** | Changes in intervention (with rationale) |
| | **9d** | Other concurrent interventions |
| **Follow-up and Outcomes** | **10a** | Clinician and patient-assessed outcomes (when appropriate) |
| | **10b** | Important follow-up diagnostic evaluations |
| | **10c** | Assessment of intervention adherence and tolerability |
| | **10d** | Adverse and unanticipated events |
| **Discussion** | **11a** | Strengths and limitations in your approach to this case—with references |
| | **11b** | Conclusions and rationale (including possible causes for outcomes) |
| **Patient Perspective** | **12** | When appropriate include the patient's perspective on this episode of care |
| **Informed Consent** | **13** | Patient informed consent is likely to be required by a journal (or your institution) prior to publication |
| **Other** | **14** | IRB approval as indicated or needed; Acknowledgement section; Competing Interests; Funding |

+1: when the item is described in the case report, 0: when the item is not reported in the case report
